# Knowledge distillation for multi-depth-model-fusion recommendation algorithm

**DOI:** 10.1371/journal.pone.0275955

**Published:** 2022-10-25

**Authors:** Mingbao Yang, Shaobo Li, Peng Zhou, JianJun Hu

**Affiliations:** 1 State Key Laboratory of Public Big Data, College of Computer Science and Technology, Guizhou University, Guiyang, China; 2 State Key Laboratory of Public Big Data, Guizhou University, Guiyang, China; 3 College of Mechanical Engineering, Guizhou University, Guiyang, China; 4 Department of Computer Science and Engineering, University of South Carolina, Columbia, South Carolina, United States of America; Asia University, TAIWAN

## Abstract

Recommendation algorithms save a lot of valuable time for people to get the information they are interested in. However, the feature calculation and extraction process of each machine learning or deep learning recommendation algorithm are different, so how to obtain various features with different dimensions, i.e., how to integrate the advantages of each model and improve the model inference efficiency, becomes the focus of this paper. In this paper, a better deep learning model is obtained by integrating several cutting-edge deep learning models. Meanwhile, to make the integrated learning model converge better and faster, the parameters of the integrated module are initialized, constraints are imposed, and a new activation function is designed for better integration of the sub-models. Finally, the integrated large model is distilled for knowledge distillation, which greatly reduces the number of model parameters and improves the model inference efficiency.

## 1. Introduction

As human beings enter the information age, people are producing a large amount of information all the time, and they also spend a lot of time browsing information on the Internet. How to let users find the information that users are interested in from the massive Internet information has become an important topic of current research. Similarly, in the aviation equipment manufacturing industry, it is also faced with the problem of how to find better upstream service providers from the massive services. The aviation equipment manufacturing cloud service platform has attracted a large number of aviation equipment manufacturing service providers to settle in. How to allow users to obtain the information they are interested in from a large number of manufacturing cloud services has become an important research content for platform developers. The recommendation algorithm is the solution important approach to this problem.

However, less research has been conducted by researchers to integrate recommendation models with various advantages to enhance model recommendation, and the integration method of manually adjusting the weight hyperparameters occupied by each depth model is inflexible and time-consuming. In addition, the integrated recommendation models are larger and the model inference speed is slower. Therefore, this study designs a new activation function to better integrate the models, and uses distillation learning to compress the integrated models. For the convenience of subsequent representation, the integrated algorithm is called MultiModel and the distilled algorithm is abbreviated as KDMRA. the main contributions of MultiModel and KDMRA are as follows:

- Add a fully connected layer, integrate the advantages of the three cutting-edge deep learning models which are DeepFM, DIN, and MMDIN models, and use the gradient descent method to update the parameters of the fully connected layer.
- In order to make the ensemble model converge faster and better and simulate the scenario where each model votes, we initialize the parameters of the model and impose limits on the parameter variation range.
- In order to make the output domain of each sub-model output as [0, 1], the output value domain of the integrated model is also [0, 1], we designed a new activation function.
- Due to the large number of parameters of the integrated model, which requires large storage space and long training time, this paper performs knowledge distillation and compression on the integrated model.

This paper uses the famous open source MovieLens dataset to validate the model. Experiments show that the integrated model has better effect than other cutting-edge deep learning recommendation models, and after the model is compressed, the model storage space is saved and the model prediction effect is improved. In addition to movie recommendation, knowledge distillation recommendation method based on multi-model fusion can also be applied to recommendation in various fields such as text, speech and video.

The rest of the paper is organized as follows: Section 2 focuses on the research work related to the recommendation algorithm. Section 3 focuses on the relevant implementation details of the model. Section 4 focuses on the data set description, baseline algorithm, relevant metrics and experimental analysis of the experiment. Finally, this paper summarizes and outlooks the experimental research.

## 2. Related works

The related work section focuses on machine learning and deep learning related recommendation work.

### 2.1. Machine learning recommendation algorithms

In order to allow users to better obtain the information they are interested in, from machine learning to deep learning, experts and scholars have proposed various methods. Cui et al. [1] proposed a method of clustering using the K-means algorithm of cuckoo search, and then using collaborative filtering for recommendation. Since user preferences change over time, Hwangbo et al. [2] proposed a user preference decay function to simulate the changes in user interests, and improved the collaborative filtering algorithm. Given that most recommendation models only predict a single rating, Nassar et al. [3] proposed a deep multi-criteria collaborative filtering model, which achieved good recommendation results. Chonghuan [4] proposed a method of clustering users first, and then using matrix factorization techniques to recommend users. Unlike most user clustering methods that use user attributes for clustering, Chen [5] uses user historical decision-making features to cluster users, and then make recommendations. In order to improve the efficiency of code development, Nguyen et al. [6] built a Persona personalized code recommendation model based on user coding preferences, experience, style, etc.

To solve the cold start problem, Nilesh et al. [7] proposed a content-based recommendation method to recommend recipes. Patra et al. [8] implemented a content-based recommendation method for recommending medical datasets. In order to solve the problem that only accurate keyword matching technology is used for recommendation in content-based recommendation, resulting in that the recommendation result field does not focus on the user’s interest field, Liu et al. [9] proposed a method that uses both keywords and popularity. recommended method. Harshvardhan et al. [10] found that there is a correlation between user access content and time, so both ratings and access time are used as inputs, and a Boltzmann machine containing time information is used to build a recommendation system. Zihayat et al. [11] proposed a news recommendation system combining utility model and probabilistic topic model, which solved the cold start problem of items. Xiao et al. [12] combined association rule-based recommendation, content-based recommendation, and collaborative filtering-based recommendation to propose a hybrid personalized recommendation system [13]. Fernández-García et al. [14] then recommend a mix of music in multiple dimensions of music genre, theme, and voice tone.

The problem of recommendation performance and incremental model updates in big data scenarios is also a hot research topic nowadays. In order to improve the disease diagnosis and treatment effect of inexperienced doctors, Chen et al. [15] clustered disease symptoms and then made recommendations. At the same time, big data technologies such as spark were used to improve the efficiency of system recommendation. To solve the problem that traditional recommender system models cannot be incrementally updated with increasing data, Khalid et al. [16] proposed a novel online recommendation algorithm that votes on active learners and hyperspheres. In order to solve the performance problem of course recommendation in big data scenarios, Zhang et al. [17] proposed a distributed association rule mining algorithm, and used Hadoop for distributed storage and spark for distributed memory computing, which improved the recommendation effect and efficiency of the algorithm. However, because machine learning models often require a lot of effort to extract features, and are limited to a relatively simple model structure, it is difficult to better fit and express massive data.

### 2.2. Deep learning recommendation algorithms

The development of deep learning and reinforcement learning [18] has brought the research on recommendation algorithms into a new era. To better capture the higher-order feature interactions in the co-occurrence matrix, Lee et al. [19] proposed a deep learning recommendation model based on convolutional neural networks. In order to improve the effect of group recommendation, Huang et al. [20] designed a multi-attention neural network to capture the internal social characteristics of groups. Since user interests shift over time, Ahmadian et al. [21] proposed a temporal reliability measure to evaluate changes in user interests. Lin et al. [22] used L0 regularization to constrain the recommendation network parameters and proposed a new convex optimization method for recommendation networks. Zhang et al. [23] integrated user location features into the recommendation model, which improved the model recommendation effect. Huang et al. [24] proposed a recommendation model that simultaneously considers taxi GPS trajectories, passenger locations, road conditions, and revenue to implement a passenger recommendation method for taxi drivers. To better push carpooling requirements for recommendations, Su et al. [25] propose recommendation models that incorporate semantic trajectories. Li et al. [26] added the topological structure of users on social networks based on the use of rating data to implement personalized recommendations for users. Lee et al. [27] proposed a self-supervised representation learning method to encode items and companies and make recommendations by computing the similarity between them. Chiu et al. [28] proposed a preprocessing model based on unsupervised learning, combined with deep learning recommendation to provide users with personalized product recommendations. In order to utilize heterogeneous information, Shi et al. [29] designed a meta-path-based random walk algorithm to generate Embedding, which is further input into the model for training and prediction.

In order to alleviate the data sparsity and cold start problems in recommender systems, Ma et al. [30] constructed a knowledge graph and made recommendations through various information such as user-item, neighbor-neighbor, etc. Ye et al. [31] obtained low-dimensional representations of various entities by constructing a knowledge graph, and then input them into a neural decomposition machine for recommendation. Since traditional recurrent neural networks can only rely on linear transformations in each session to train recommendation models, Gwadabe et al. [32] propose a graph neural network-based recommender system that simultaneously uses non-sequential interactions and sequential The interactive information is used for model training, which improves the model recommendation effect. To solve the data sparsity and cold-start problems, Li et al. [33] investigated the construction and mining of higher-order semantic information in knowledge graphs and applied them to scholar recommendations. To solve the problem that GNNs are difficult to capture the implicit information in the interaction direction dimension, Chang et al. [34] propose a novel graph neural network for reciprocal recommendation. Since a similar group of users may also have some different interests, Ali et al. [35] used Pearson similarity to assign users to multiple interest groups, and combined group information and other information for recommendation, which improved the recommendation effect. Since customer reviews play an important role in recommendation decisions, Karthik et al. [36] proposed a fuzzy recommendation system based on sentiment analysis and customer interests. Moscato et al. [37] identify the user’s emotion and recommend music to the user according to the user’s emotion. Abbasi-Moud et al. [38] performed sentiment analysis and semantic clustering on user reviews, extracted user preferences, and used these user preferences to recommend users. To address the cross-domain problem in recommendation using sentiment analysis, Osman et al. [39] proposed a domain-adaptive recommendation algorithm using contextual information.

In order to protect user privacy, Qi et al. [40] designed an enhanced locality-sensitive hash recommendation algorithm. Gong et al. [41] also improved the local-sensitive hashing algorithm to protect user privacy from multiple dimensions. In order to protect data privacy, Chen et al. [42] proposed a new privacy-preserving method for recommender systems based on context awareness. Li et al. [43] used geographic information to effectively improve the system recommendation effect. To protect user privacy, Huo et al. [44] proposed a location-based privacy-preserving algorithm, which was integrated into the recommender system, and achieved a good balance between privacy and recommendation accuracy. In order to explain the recommendation results, Hsu et al. [45] proposed an explanatory recommendation system based on a knowledge graph to recommend funds.

## 3. KDMRA

**Fig 1:**
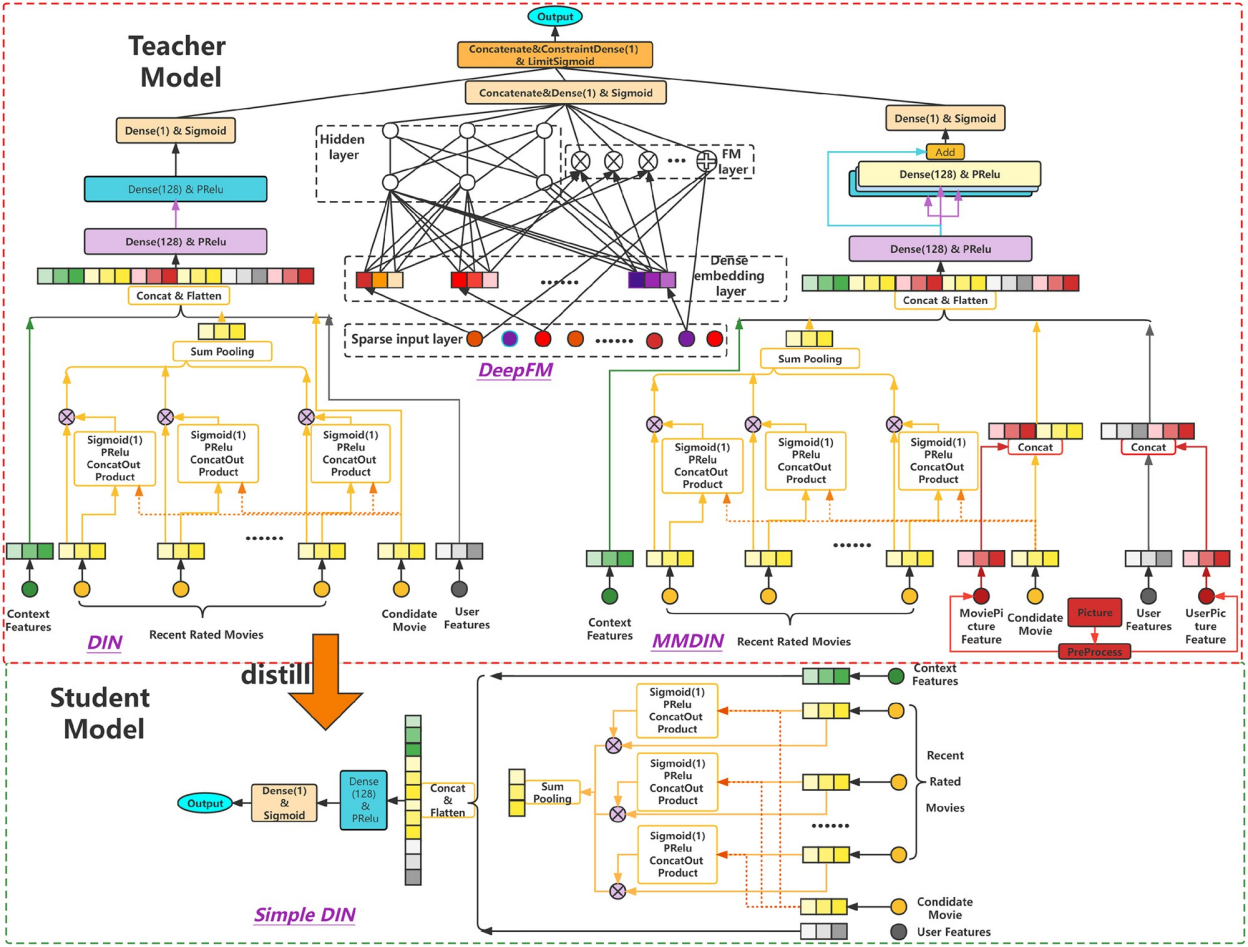
The structure of the proposed algorithm.

The overall implementation process of KDMRA is shown in Fig 1. The algorithm mainly includes the teacher model part (the upper part of the figure) and the student model part (the lower part of the figure). The teacher model mainly integrates three mainstream recommendation algorithms, namely DIN (left), DeepFM (middle) and MMDIN (right). The student model is implemented using a simple shallow DIN model. Each module is described in detail below.

In addition, the notations used in this paper and their meanings are shown in Table 1.

**Table 1:** Annotations.

Notations	Descriptions
V	Current parameter value
P	Previous parameter value
R	Rate of change
Vmin	Minimum value for parameter qualification
Vmax	Maximum value for parameter qualification
b	Initial Factor
k	Positive adjustment factor
Lsum	Total loss
L1	Loss between soft value and model predicted value
L2	Loss between true value and predicted value
α	Distillation factor
TP	True Positive
TN	True Negative
FP	False Positive
FN	False Negative
F1-Score	Summed average of precision and recall
TPR	True Positive Rate
FPR	False Positive Rate
G-Mean	Geometric mean of positive sample recall and negative sample recall
PR-AUC	The area under the PR curve
ROC-AUC	The area under the ROC curve

### 3.1. DIN module

The advantage of DIN is that it introduces an attention mechanism to avoid treating items that need to be rated equally. The main idea is to use the 5 movies recently rated by the user to do the outer product with the current candidate movies and weighted pooling to obtain the User’s recent points of interest. As shown in the model in the upper left corner of Fig 1, the original input sparse features and non-numeric data are encoded to form dense feature vectors. Then, take the outer product of the current movie and the last 5 movies respectively (as shown in the yellow-edged parts in the upper left corner of Fig 1), and then connect them for PRelu and sigmoid activation to get the similarity between the current movie and the recently rated movie, and weight the similarity to each recently rated movie. above, do summation pool-ing. Finally, it is connected with user features and scene features, and is fitted and expressed through a multi-layer neural network (as shown in the three parts in the upper left corner of Fig 1) to obtain the final scoring result.

### 3.2. DeepFM module

DeepFM improves the Wide&Deep model, changing the wide part to the FM part, so that the features can be combined and crossed, and then the item scores can be predicted. The DeepFM model structure is shown in the middle area of the upper part of Fig 1. Numerical features can be directly input to the next layer for operation, while non-numerical features are densified to obtain corresponding embedded representations. Some of the features are directly combined with feature crossover (as shown in the FM layer in Fig 1), and finally connected to the fully connected layer for activation and output; the other part is subjected to complex fitting and expression through a multi-layer neural network (as shown in the Hidden layer in Fig 1), and finally connected to the output layer. DeepFM not only ensures that the model has strong memory ability and generalization ability, but also enables the model to have the ability to predict the score by combining features.

### 3.3. MMDIN module

On the basis of DIN, the MMDIN module introduces the image features of items to predict user scores, and adds a multi-head mechanism to enable the model to extract features from different dimensions.

The MMDIN model is mainly divided into a multi-modal module, an attention module and a multi-layer neural network module. The multi-modal module (as shown in Fig 1 MMDIN module in red parts) is mainly responsible for extracting the color features of the image; the attention mechanism module (as shown in the yellow part of the MMDIN module in Fig 1) is responsible for extracting items that the user may be interested in from the items recently rated by the user. The multi-layer neural network module mainly adopts the Reset structure to alleviate the problem of gradient disappearance when the number of model layers becomes deeper, making it difficult for the scoring prediction to become worse as the model becomes deeper. At the same time, the multi-head mechanism (as shown by the trigeminal arrow in the MMDIN module in Fig 1) is adopted in the multi-layer neural network, so that the model can better extract features from the data from multiple dimensions.

### 3.4. Integrated learning module

The integrated learning module is mainly responsible for integrating the advantages of the DeepFM, DIN, and MMDIN models (as shown in the orange parts in the top half of Fig 1), so that the model has a better prediction effect, and also makes the algorithm more robust and stable. The ensemble learning module uses the fully connected layer to perform weighted voting on the prediction results of the three models to obtain the final prediction result. Among them, the weights are adaptively adjusted by the gradient descent method. In order to simulate the voting scenarios of each model, so that the model can converge better and faster, the parameters of the fully connected layer are specified and initialized, constraints are imposed, and the rate of change is set to specify the rate of parameter change. The parameter calculation method is as shown in Eq 1 shown. Among them, V represents the current parameter value, P represents the previous parameter value, R represents the rate of change, V_min_ represents the minimum value limited by the parameter, and V_max_ represents the maximum value limited by the parameter.


$$V=(1-R{)}^{*}P+R^{*}\left\{\begin{array}{cc} V_{\text{min}} & P<V_{\text{min}}\\ P & V_{\text{min}}\leq P\leq V_{\text{max}}\\ V_{\text{max}} & P>V_{\text{max}} \end{array}\right.$$ (1)

In addition, in order to make the output range of each sub-model be [0, 1], the output range of the integrated model is also [0, 1], that is, the final output score range, which is more in line with the actual change scenario, we study A new excitation function is designed, and the calculation method of the excitation function is shown in formula 2.


$$y=\frac{e^{\text{kx}}}{e^{\text{kx}}+b}$$ (2)

**Fig 2:**
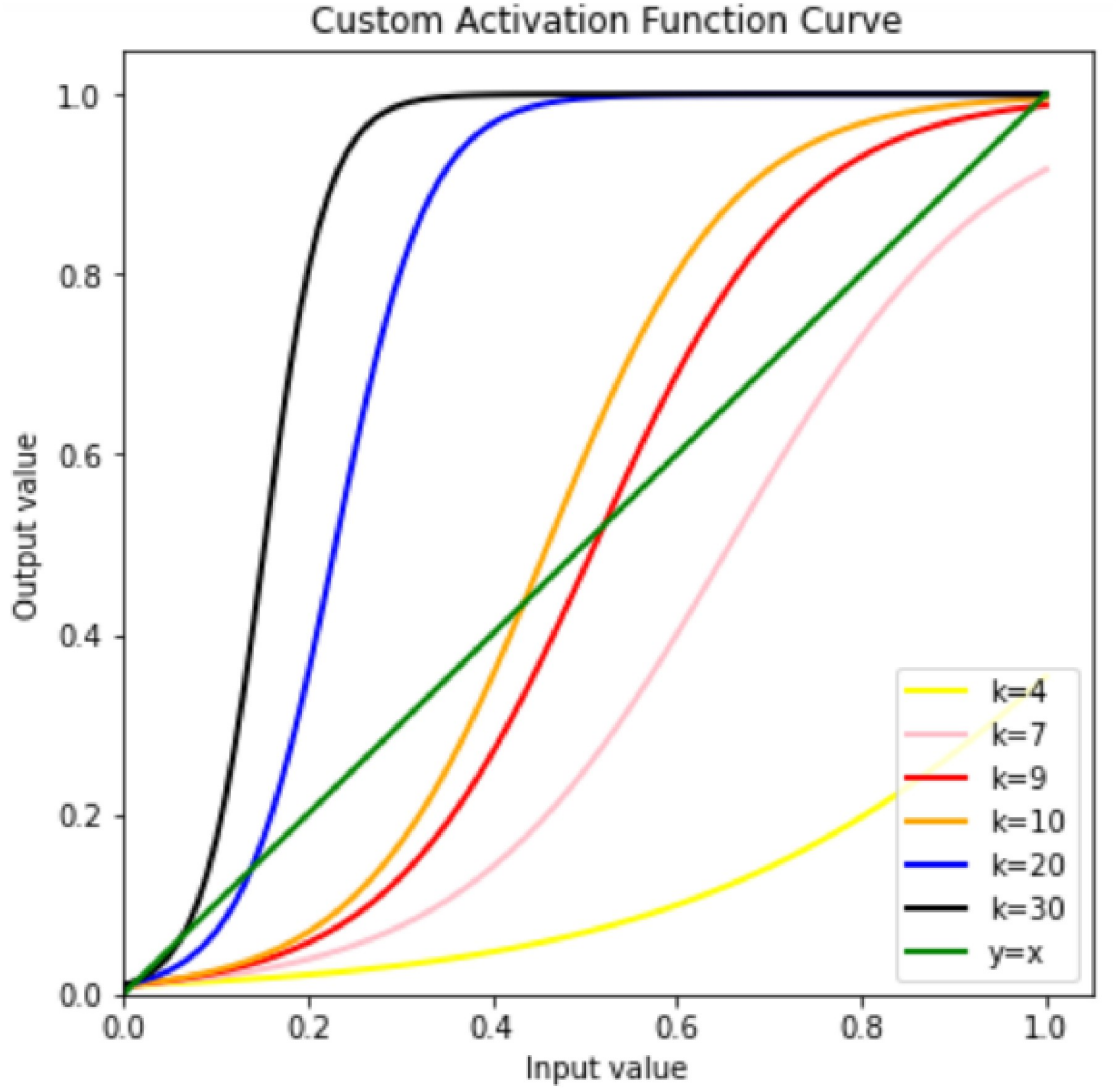
Custom activation function change curve with the k value changing.

Among them, x is the input, y is the output, b is the initial factor, and k is the proportional adjustment coefficient. The closer the curve is to y = x, the better the activation effect of the activation function. Set the initial factor b = 100, adjust k, and get the activation effect of the activation function as shown in Fig 2. It can be intuitively obtained from Fig 2 that ask increases, the degree of the curve close to the straight line y = x becomes smaller and smaller. After reaching the minimum value, ask increases, the curve begins to move away from the straight line y = x. This paper takes b = 100, k = 9.24 for experiments.

### 3.5. Student module

The student module is mainly implemented with a simple shallow DIN structure. The advantage of knowledge distillation lies in the use of soft labels to guide the convergence of students’ models, enabling students to make accurate predictions on samples they have not seen before. The loss function design of the distillation model is shown in Eq 3. Among them, L_sum_ represents the total loss, L_1_ represents the loss between the soft value and the model predicted value, L_2_ represents the loss between the true value and the predicted value, and α is the distillation coefficient.


$$L_{\text{sum}}=L_{1}\alpha +L_{2}(1-\alpha )$$ (3)

At this point, the KDMRA algorithm is introduced, and its main implementation process is sorted out as shown in Algorithm 1.

**Algorithm 1**: KDMRA

 **Input**: user information, item information, user item rating

 **Output**: Item score prediction

1 Initialize parameters

2  1. User features, item features and user-item relationship features pre-processing.

 2. The processed features are input into DIN, DeepFM, and MMDIN models for prediction.

  2.1 The DIN model calculates the current candidate movie attention score from the user’s recently viewed movies, weighted pooling to the current movie code, and input to the MLP layer along with user features, movie features and scene features for fitting and output.

  2.2 After thickening the ID-type features, the DeepFM model cross-combines the shallow features and connects them to the fully connected layer, while the other part performs complex crossover by MLP and then connects to the fully connected layer for output.

  2.3 MMDIN adds the use of picture-related features to DIN and uses multiple header Heads to extract the features, which are finally output to the MLP layer for fitting and output.

3  3. Integration: Integration of multiple models using custom activation functions that map both the input and output domains to the [0,1] range, simulating a voting scenario with dynamic weights to map the inputs to the outputs.

 4. Train and save models for evaluation.

4 Return

## 4. Experiments

### 4.1. Dataset description

This paper uses the open-source MovieLens dataset to verify the model effect. The distribution information of the dataset is shown in Table 2.

**Table 2:** Dataset distribution.

	Training dataset	Testing dataset	Positive rate on training dataset	Positive rate on testing dataset	Total Num
**MovieLens**	4,039,104	1,009,235	54.44%	54.48%	5,048,339

The MovieLens dataset mainly includes a movie dataset and a user rating dataset for movies. Among them, the user movie rating dataset mainly includes attributes such as user id, movie id, the user’s rating of the movie, and the timestamp of the rating; the movie dataset mainly includes movie id, title, movie style, release year, and movie poster. As shown in Table 2, all the reviews of the first 1000 movies are selected, and after cleaning and preprocessing the movie dataset, the dataset has a total of 5,048,839 rating records, of which 20% are selected for test verification. Among them, in the training set and the test set, the proportion of user praise is 54.44% and 54.48% respectively, indicating that the distribution of the test set data and the training set data is basically the same.

### 4.2. Baseline algorithm

In the following experiments, we use five cutting-edge deep learning recommendation algorithms as benchmark algorithms for experimental comparison and analysis.

- NeuralCF: NeuralCF uses a multi-layer neural network to replace the simple interoperability layer in matrix decomposition, and at the same time solves the limitation that only rating information can be used in matrix decomposition so that user-side features and item-side features can be better crossed.
- Embedding MLP: It mainly includes a feature input layer, Embedding layer, Stacking layer, multi-layer residual network layer, and scoring output layer. The model mainly realizes the simultaneous input of numerical features and non-numerical features into the model for training, and uses residual connections to alleviate the problem of network gradient disappearance.
- DeepFM: On the basis of the WideDeep model, the FM layer is added, so that the model has a better recommendation effect in scenarios where the combined features have a great influence on the prediction results.
- DIN: The e-commerce advertising recommendation model released by Ali, which introduces an attention mechanism, takes the outer product of the user’s recently rated products and candidate products to obtain the attention score, and then weights it to the recently rated products and performs summation and pooling. It is finally input into the MLP model for scoring prediction.
- MMDIN: On the basis of DIN, multi-head mechanism and multi-modal method are added. The multi-head mechanism enables the model to extract features from data from multiple dimensions. The multi-modal method mainly mines image features to train the model, which enriches the feature library that the model can use.

### 4.3. Experiment setup and evaluation index

This experiment runs in Ubuntu 18.04 and Cuda 11.1 environment, which is configured with 8-core CPU, 16G memory, GeForce RTX 2060 super graphics card, and 8G memory size. Developed with Python 3.8.1, Tensorflow 2.5 and Spark 2.4.3.

The experimental evaluation mainly uses comprehensive indicators such as PR-AUC, ROC-AUC, and F1-Score, as well as single indicators such as Accuracy, Recall Rate, and Precision. The formulas for precision and recall are shown in Eqs (4) and (5).


$$\text{Pr}ecision=\frac{TP}{TP+FP}$$ (4)

$$\text{Re}call=\frac{TP}{TP+FN}$$ (5)

The F1-Score is the summed average of the precision rate and recall rate. F1-Score is the summed average of precision and recall, which is a comprehensive evaluation index of precision and recall, and its calculation formula is shown in Eq (6).


$$F1-Score=\frac{2*\text{Pr}ecision*\text{Re}call}{\text{Pr}ecison+\text{Re}call}$$ (6)

G-Mean is also a comprehensive evaluation index of the recommendation model, which has a better evaluation effect on the recommendation scenario in the case of data imbalance, and its calculation formula is shown in Eq (7).


$$G-M\text{ean}=\sqrt{\frac{TP}{TP+FN}+\frac{TN}{TN+FP}}$$ (7)

The true positive rate (TPR), false positive rate (FPR), and equilibrium accuracy were calculated as shown in Eqs (8), (9) and (10), respectively.


$$TPR=\frac{TP}{TP+FN}$$ (8)

$$FPR=\frac{FP}{FP+TN}$$ (9)

$$\text{Balanced}\;\text{Accuracy}=\frac{TPR+TNR}{2}$$ (10)

The PR curve is a combined dynamic assessment of precision and recall, while the ROC curve is a combined dynamic assessment of true-positive and false-positive rates. Two metrics, PR-AUC and ROC-AUC, indicate the magnitude of the area enclosed by the PR and ROC curves and the coordinate axis, respectively. In this experiment, the Adam optimizer is used to optimize each model.

### 4.4. Evaluation of experimental results

#### 4.4.1. Comparison of key evaluation indicators for recommendation algorithms

In order to evaluate the pros and cons of the model, this study uses ROC-AUC, PR-AUC, FScore and other comprehensive indicators to comprehensively evaluate the model, and uses single evaluation indicators such as Precision, Recall, Accuracy, and Loss to evaluate the model unilaterally. Among them, the industry-recognized and most used indicator is the ROC-AUC indicator. This experiment is carried out on the MovieLens, and the evaluation results are shown in Table 3 below.

**Table 3:** Comparison of key evaluation indicators for recommendation algorithms.

Methods	ROC-AUC	PR-AUC	F1-Score	Precision	Recall	Accuracy	Balanced Accuracy	Loss	Evaluation Time(s)
**NeuralCF**	0.7319	0.7537	0.7358	0.6738	0.7701	0.6717	0.6612	0.6030	41
**EmbeddingMLP**	0.7610	0.7786	0.7483	0.7060	0.7576	0.6961	0.6906	0.5792	**40**
**DeepFM**	0.7799	0.7947	0.7614	0.7134	0.7872	0.7118	0.7041	0.5598	41
**DIN**	0.7897	0.8028	0.7671	0.7064	**0.8263**	0.7183	0.7083	0.5582	42
**MMDIN**	0.8010	0.8156	0.7724	0.7334	0.7877	**0.7284**	0.7222	**0.5370**	46
**Multi-Model**	**0.8018**	**0.8171**	**0.7736**	0.7549	0.7354	0.7258	0.7226	0.6250	238
**KDMRA**	0.8007	0.8149	**0.7736**	**0.7577**	0.7262	0.7243	**0.7231**	0.5690	42

**Fig 3:**
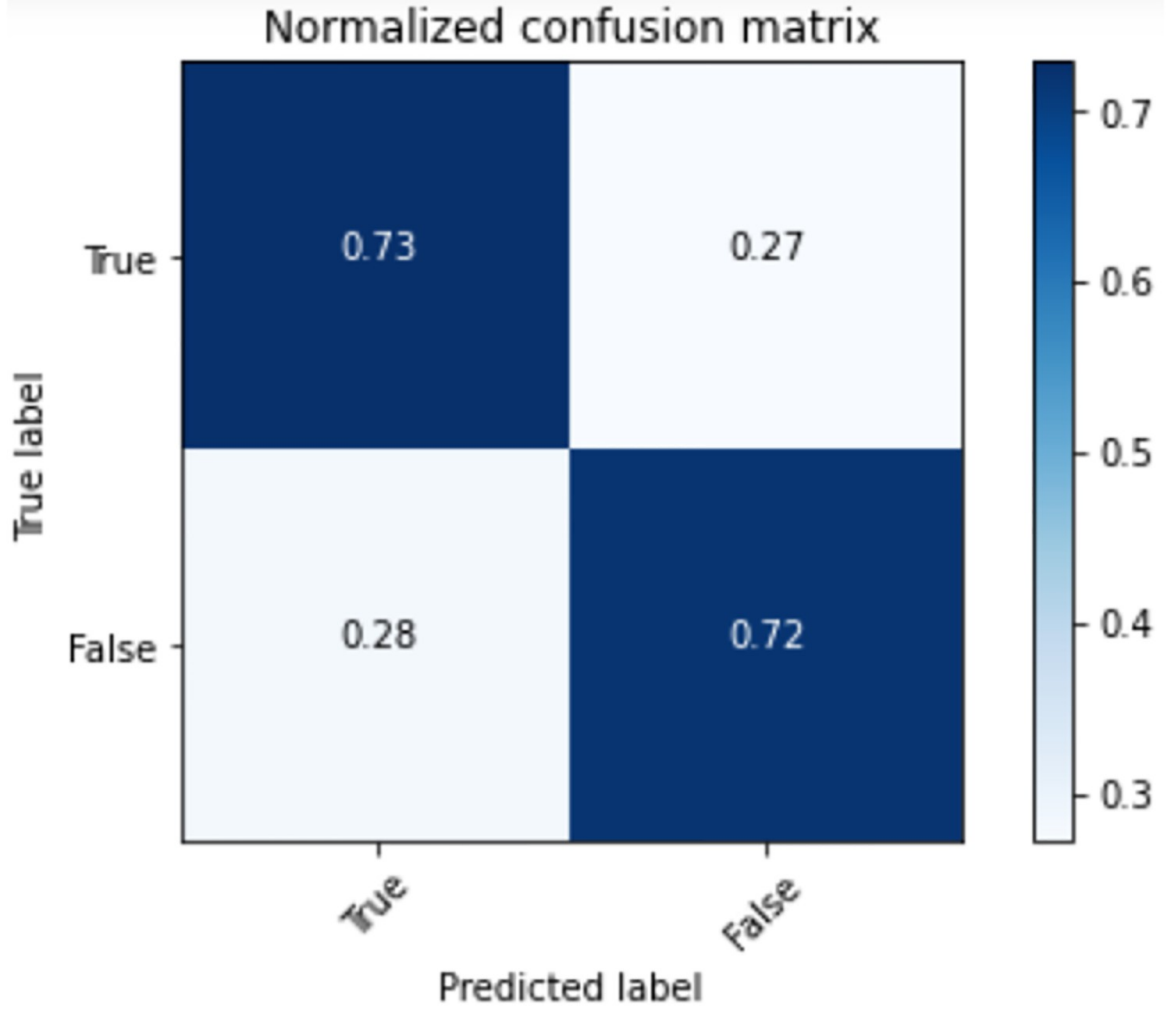
Confusion matrix of KDMRA.

It can be seen from Table 3 that in the experimental evaluation results of MovieLens, the multimodel integration algorithm MultiModel has the highest value in the three comprehensive evaluation indicators ROC-AUC, PR-AUC and F1-Score, and the single evaluation indicators Precision, Recall, Accuracy, Balanced Accuracy, Loss, KDMRA, MMDIN and DIN models have their own strengths. As a compressed version of the multi-model ensemble model MultiModel, KDMRA also has good results. In addition, in the evaluation of 1,009,235 test data, the MultiModel model took 238s, while KDMRA used only 42s, significantly reducing the inference time required by the multi-model fusion algorithm. It can be seen that on the MovieLens dataset, the comprehensive performance ranking of each model is: MultiModel > MMDIN > KDMRA > DIN > DeepFM > Embedding MLP > NeuralCF. Among them, the confusion matrix of KDMRA is shown in Fig 3. As can be seen from Fig 3, the KDMRA accuracy is basically consistent with the accuracy in Table 3.

#### 4.4.2. Precision and recall rate curve of each model

**Fig 4:**
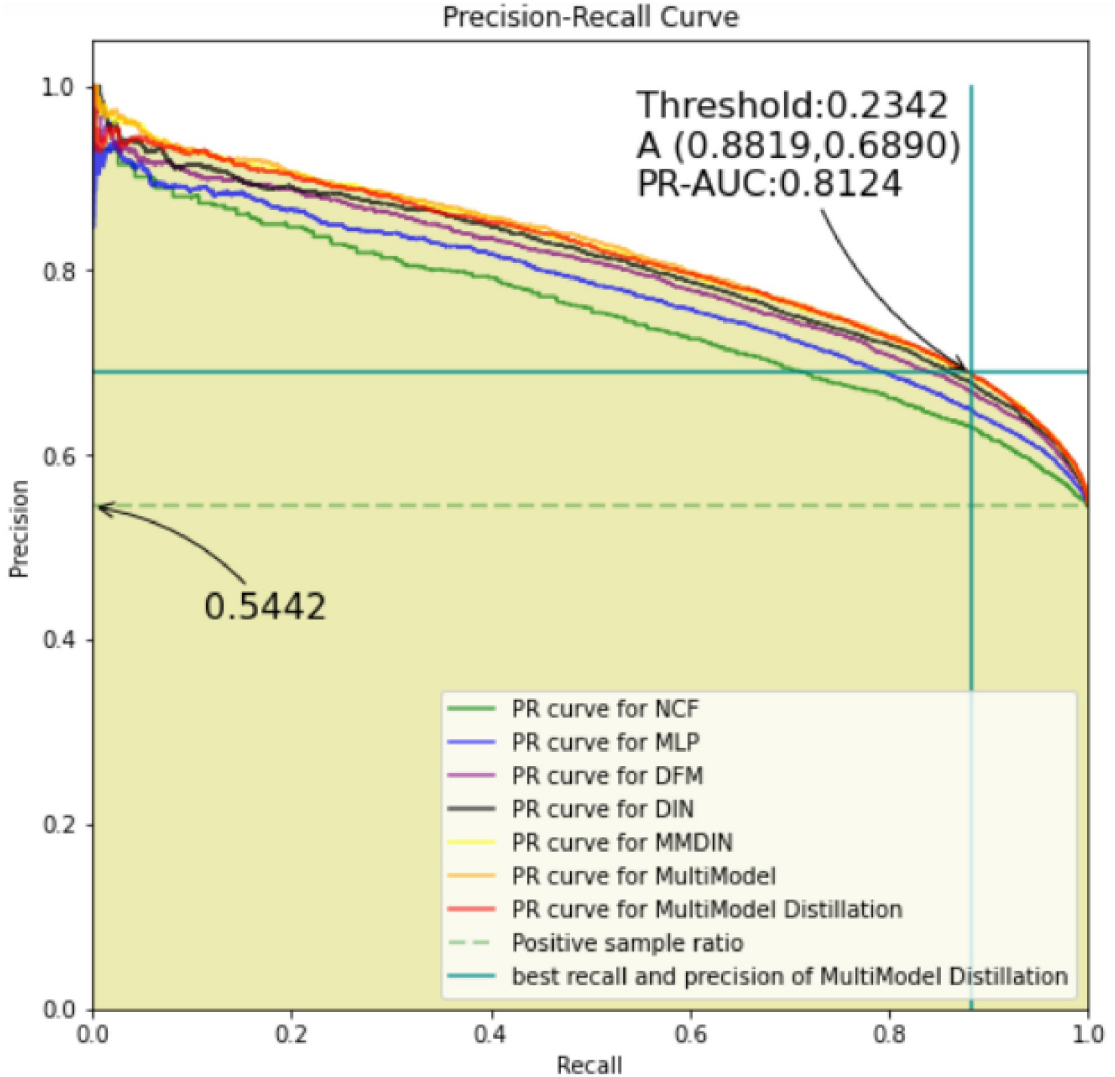
The precision and recall rate curve of each model on the MovieLens dataset.

In order to analyze the recommendation effect of each model more intuitively, in this experiment, after shuffling the MovieLens datasets, 20,000 pieces of data were randomly selected for evaluation and PR curve was drawn, as shown in Fig 4.

In the PR curve, the larger the area under the curve, the better the model recommendation effect. It can be seen from Fig 4 that among the 20,000 pieces of rating data extracted this time, the favorable rate is 0.5442, which is equal to the favorable rate on the entire dataset (as shown in Table 2). The uppermost curve in Fig 4 is the yellow curve, which represents the curve where the MultiModel is located. This is followed by the red and yellow curves, representing the curves where MMDIN and KDMRA are located, respectively. The overall ranking of model recommendation effects is: MultiModel > MMDIN > KDMRA > DIN > DeepFM > Em bedding MLP > NeuralCF. Among them, when Threshold is 0.2342, the KDMRA model achieves the best precision rate and recall rate, which are 0.6890 and 0.8819, respectively, and the corresponding PR-AUC value is 0.8124 (the formula for calculating each indicator is shown in subsection 4.3).

It can be seen from Fig 4 that the MultiModel algorithm has a better recommendation effect than other models on the MovieLens dataset. The model compressed by the knowledge distillation method also has a good recommendation effect.

#### 4.4.3. Receiver operating curve of each model

**Fig 5:**
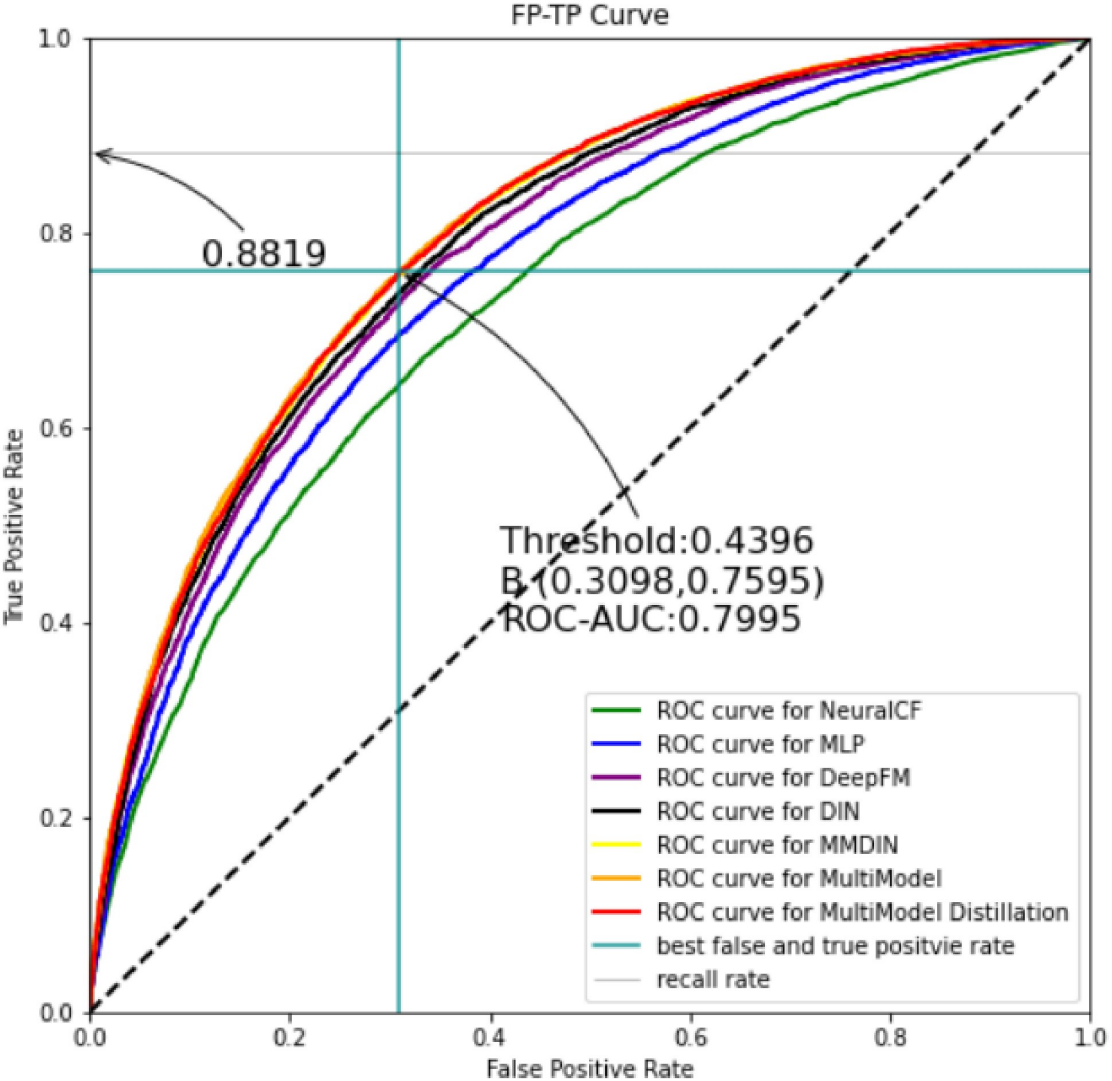
Receiver operating curves of each model on the MovieLens dataset.

The ROC curve is a recognized indicator in the recommendation system that can comprehensively reflect the comprehensive recommendation effect of the recommendation model. Like the PR curve, the larger the area under the ROC curve, the better the model recommendation effect. The recommendation effects of each model on the MovieLens datasets are shown in Fig 5.

In Fig 5, the three curves of yellow, red and orange basically overlap, representing the curves of MultiModel, MMDIN and KDMRA respectively, and the yellow curve is slightly higher than the red curve, followed by the orange curve. The following are the curves of black, purple, blue, and green, respectively ghostwriting the curves of DIN, DeepFM, Embedding MLP, and NeuralCF, that is, the recommended effect size order is: MultiModel > MMDIN > KDMRA > DIN > DeepFM > Embedding MLP > NeuralCF. When Threshold is 0.1658, the true positive rate and false positive rate of KDMRA model are 0.8646 and 0.3270, respectively, the ROC-AUC value at this time is 0.8362, and the recall rate is 0.8646 (the formula for calculating each indicator is shown in subsection 4.3).

It can be seen from Fig 5 that the MultiModel model has a better comprehensive recommendation effect than other models, and the KDMRA model after knowledge distillation also has a better effect.

#### 4.4.4. Model intuitive prediction effect analysis

**Fig 6:**
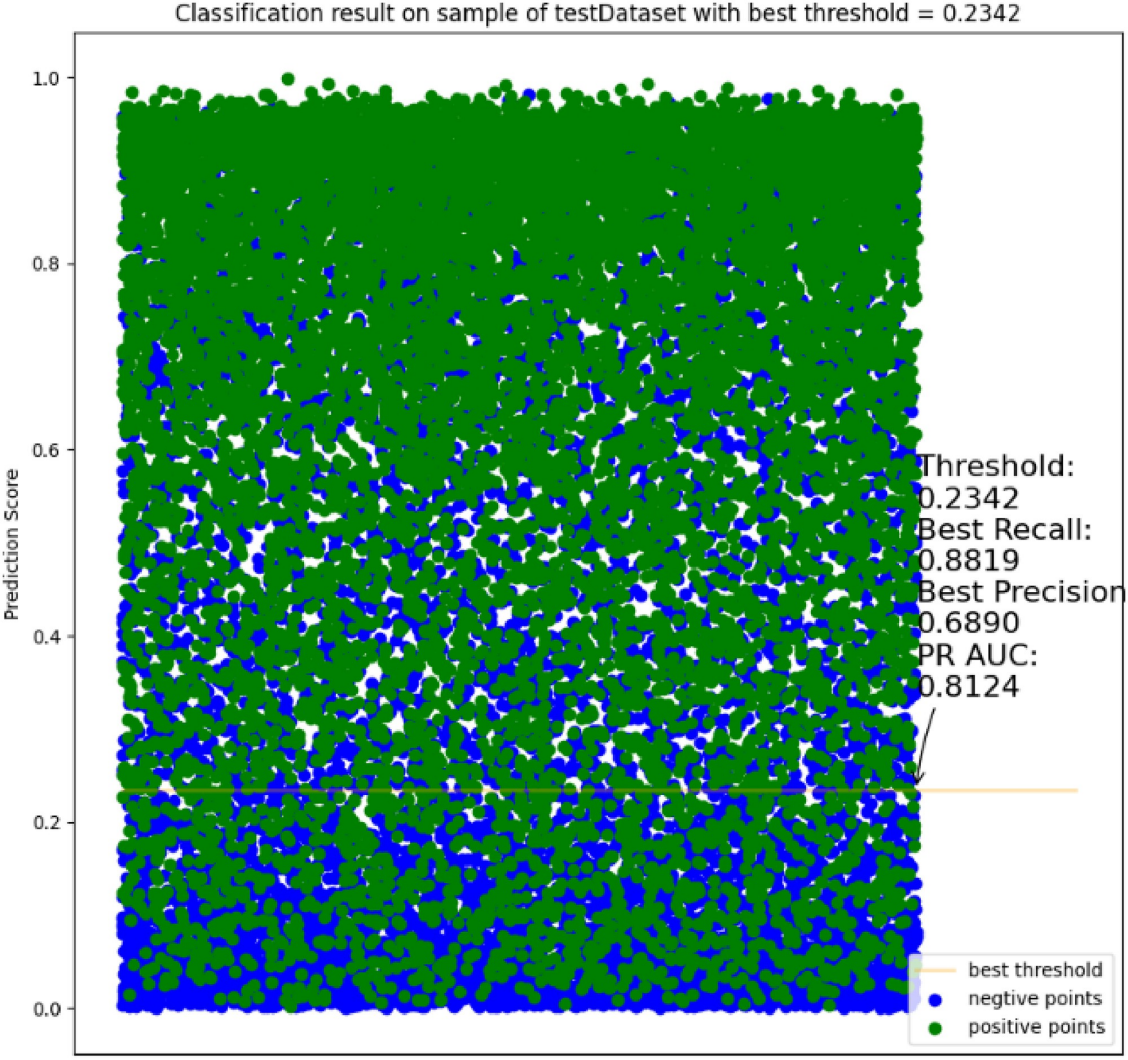
Model intuitive classification effect on the MoiveLens dataset.

In order to show the model prediction effect more intuitively, in this experiment, after shuffling the MovieLens datasets, 20,000 pieces of data are randomly selected for scoring prediction, and then the scatter plot is drawn as shown in Fig 6. Among them, the ordinate represents the score prediction result, and the abscissa represents any floating point number between 0.0 and 1.0.

It can be seen from Fig 6 that the green points represent the points that the user actually rated as positive, and these points are mainly distributed in the upper part of the image, that is, the KDMRA model predicts a relatively high score. The blue dots represent the points where the user’s actual rating is negative. These points are mainly distributed below the image, that is, the rating predicted by the model is also relatively low, which indicates that the rating prediction result of the model is reliable. Among them, when the threshold is 0.2342, the model achieves the best recall rate and precision rate, which are 0.8819 and 0.6890, respectively, and the PR-AUC value at this time is 0.8124 (the formula for calculating each indicator is shown in subsection 4.3).

#### 4.4.5. Effectiveness of MultiModel and its distillation model

**Fig 7:**
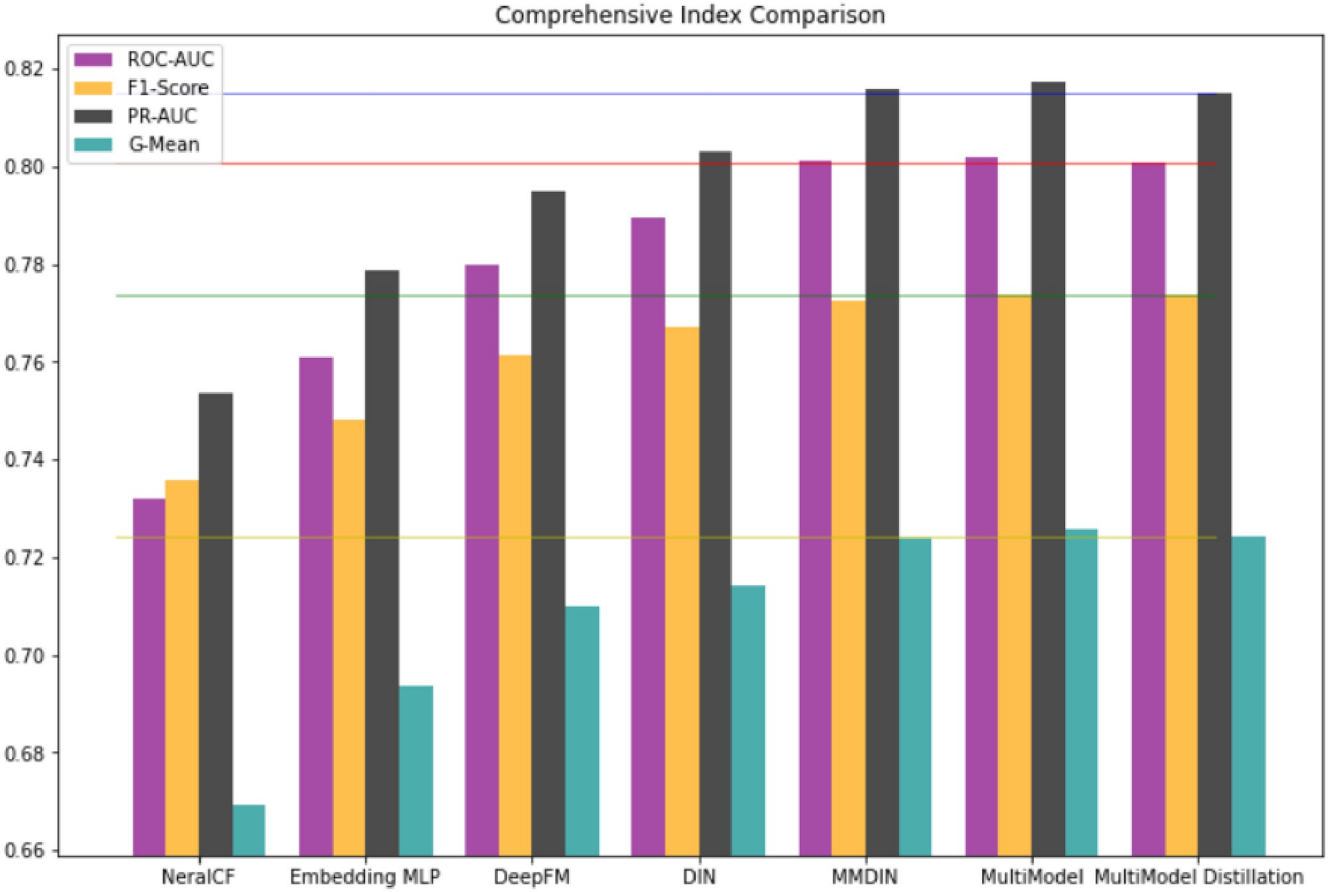
Comparison of comprehensive indicators of each model on the MovieLens dataset.

In order to further conduct an intuitive and comprehensive evaluation of MultiModel and its distillation model, this experiment draws a columnar comparison chart of the comprehensive evaluation indicators of each model for comparison, as shown in Fig 7.

It can be seen intuitively from Fig 7 that on the whole, the MultiModel model has high values in various comprehensive indicators such as ROC-AUC, F1-Score, PR-AUC, and G-Mean (the formula for calculating each indicator is shown in subsection 4.3). The compressed KDMRA model also has higher values.

In summary, through the PR curve, receiver operating curve and comprehensive evaluation index of the model, it can be concluded that MultiModel has a better recommendation effect than other models, and its distilled KDMRA model also has a better recommendation effect.

## 5. Conclusions and future work

In this study, we integrate the advantages of several models to obtain a model with better recommendations. In order to make the model converge faster and better, we initialize and impose constraints on the model parameters. In addition, we design a new activation function to better simulate the sub-model voting scenario. Finally, to reduce the number of model parameters and improve the model inference speed, we distill the integrated large model with the knowledge to improve the recommendation efficiency. In conclusion, the multi-model fusion knowledge distillation recommendation algorithm effectively improves the system recommendation effect and recommendation system, and the method can be applied in various fields such as e-commerce recommendations, advertising recommendations, and long and short video recommendations.

In the future, we plan to continue in-depth research on recommender systems in the following aspects. First, study the recommendation of lifelong learning to improve the recommendation effect of the model after updating the model parameters with incremental data. Then, we study the effect of the variable-length self-attention mechanism on the improvement of recommendation effect. Finally, we study multi-modal reinforcement learning recommendations by simulating human learning by interacting with the environment.

## Supporting information

S1 Text Supporting data for all the figures and tables in this text. (DOCX)
