# Supplementary material for: Knowledge distillation for multi-depth-model-fusion recommendation algorithm
Source: PLoS One. 2022 Oct 25;17(10):e0275955. doi: 10.1371/journal.pone.0275955 (PMC9595540; doi:10.1371/journal.pone.0275955)
Supplement: S1 Text — (DOCX) [file pone.0275955.s001.docx]

## Supporting information

[The MovieLens dataset](https://grouplens.org/datasets/movielens/). Supporting data for all the figures and tables in this text.
